# A descriptive survey study of violence management and priorities among psychiatric staff in mental health services, across seventeen european countries

**DOI:** 10.1186/s12913-017-1988-7

**Published:** 2017-01-19

**Authors:** Seamus Cowman, Anna Björkdahl, Eric Clarke, Georgina Gethin, Jim Maguire, European Violence in Psychiatry Research Group (EViPRG), Christoph Abderhalden, Adriana Miha, Roger Almvik, Hulya Bilgin, Patrick Callaghan, Thanos Douzenis, Frans Fluttert, Irina Georgieva, Jacob Hvidhjelm, Regina Ketelsen, Peter Lepping, Maria Isabel Dias Marques, Vesna Petrovic, Jon Snorrason, Bart Thomas

**Affiliations:** 1 Royal College of Surgeons in Ireland- Bahrain, P.O. Box 15503, Adliya, Bahrain; 2 Department of Clinical Neuroscience, Centre for Psychiatric Research, Karolinska Institute, Stockholm, Sweden; 3 Royal College of Surgeons in Ireland, Dublin, Ireland; 4 National University Ireland, Galway, Ireland; 5 Athlone Institute of Technology, Dublin, Ireland

**Keywords:** European mental health policy, Violence management, Coercion, eDelphi, Education and research priorities

## Abstract

**Background:**

In mental health services what is commonplace across international frontiers is that to prevent aggressive patients from harming themselves, other patients or staff, coercive measures and foremost, violence management strategies are required. There is no agreement, recommendations or direction from the EU on which measures of coercion should be practiced across EU countries, and there is no overall one best practice approach.

**Methods:**

The project was conceived through an expert group, the European Violence in Psychiatry Research Group (EViPRG). The study aimed to incorporate an EU and multidisciplinary response in the determination of violence management practices and related research and education priorities across 17 European countries. From the EVIPRG members, one member from each country agreed to act as the national project coordinator for their country. Given the international spread of respondents, an eDelphi survey approach was selected for the study design and data collection. A survey instrument was developed, agreed and validated through members of EVIPRG.

**Results:**

The results included a total of 2809 respondents from 17 countries with 999 respondents who self-selected for round 2 eDelphi. The majority of respondents worked in acute psychiatry, 54% (*n* = 1511); outpatient departments, 10.5% (*n* = 295); and Forensic, 9.3% (*n* = 262). Other work areas of respondents include Rehabilitation, Primary Care and Emergency. It is of concern that 19.5% of respondents had not received training on violence management. The most commonly used interventions in the management of violent patients were physical restraint, seclusion and medications. The top priorities for education and research included: preventing violence; the influence of environment and staff on levels of violence; best practice in managing violence; risk assessment and the aetiology and triggers for violence and aggression.

**Conclusion:**

In many European countries there is an alarming lack of clarity on matters of procedure and policy pertaining to violence management in mental health services. Violence management practices in Europe appear to be fragmented with no identified ideological position or collaborative education and research. In Europe, language differences are a reality and may have contributed to insular thinking, however, it must not be seen as a barrier to sharing best practice.

## Background

In the modern Europe with frontiers continuing to expand, EU Directives and regulation are promoted as a means of unifying and enforcing best practice in many diverse fields such as agriculture, education and social services. However, EU Directives and regulation on mental health remains light touch and without any overall agreement on best practice and standards. Such a position is difficult to comprehend, given that in agriculture the use of land and the health and welfare of farm animals and fishing is regulated, yet in mental health services, a vital and controversial human intervention such as coercion and violence management is devoid of EU direction on best practice. In the European region there is much variance in the level of investment and available services for the care and treatment of mentally ill persons, one of our most vulnerable groups in society. Mental health problems may account for nearly 20% of the total burden of ill health in Europe, yet it has been estimated that some mental health budgets are less than 5% of the health budget, with no uniformity across Europe [1]. Across the EU, psychiatric care environments range from outdated overcrowded Dickensian institutions to purpose built modern units. High staff turnover and problems with recruitment and retention are a salient feature of psychiatric care in Europe [2]. There are distinctive differences between Western and Eastern Europe in terms of developments in mental health services. In recent decades there have been major political changes in Central and Eastern Europe with social and political revolutions leading to radical changes in health care systems. The resulting greater transparency has focused attention on levels of abuse and misuse of the mental healthcare systems, including their past use to discipline and punish social dissidents.

In some areas of health care there are EU Directives implemented to govern standards; most notably since the 1970s there are EU directives for the undergraduate education and training of medical doctors [3] and directives about specialist training in psychiatry from the European Union of Medical Specialists [4] (EUMS). European Directive for nursing also were introduced [5]. A European survey reviewing EUMS indicated that despite progress there were significant differences between training centres in different countries and training in Europe was heterogeneous with differing standards across countries [6].

In the light of such direction on education and training of health professionals, the lack of an EU policy position on standards of care and clinical practice in mental health care is difficult to comprehend. The lack of a unifying direction and strategy on the treatment and management of mentally ill people has often resulted in individuals and families appealing to the EU Court of Human Rights to resolve outstanding issues related to the mentally ill. Article 3 of the Human Rights Act is the only absolute European Convention right and it implies that - no one shall be subjected to torture or to inhuman or degrading treatment or punishment. There is a developing range of case law evolving in relation to Article 3 and issues of psychiatric treatment [7].

The management of acute mentally ill people, during psychiatric crisis, poses particular difficulties for mental health professionals, given the increased risk of violent behavior. This paper outlines approaches to the management of violence across the mental health services of 17 European countries, and the education and research priorities as reported by staff working in those services. The results provide a substantial basis to guide best practice, education and research for violence management in mental health services across Europe.

### Managing violence in mental health services

Historical accounts of mental health services, document a prominent evolving role for staff in violence management in the traditional psychiatric hospital setting. In those early days a major element of the mental health role involved the enforcement of rules [2]. The evolving mental health services witnessed the demise of traditional mental health hospitals with a policy of more open community services. There continues to be an escalation in violence, and it has been identified, [8] that almost a quarter of patients admitted to hospital reported thoughts of violence directed at specific individuals. The relationship between the use of illegal drugs and violence is increasing, and almost two decades ago, the illicit use of drugs among psychiatric inpatients as a major security issue, was highlighted [9].

In mental health, crises are created through psychotic episodes, suicidal and self-harming behavior, alcohol and drug abuse, attempted absconding, and sometimes over-stimulation by the ward environment. Such factors may create a danger to staff and/or other patients and the challenge for staff is to maintain their own safety and that of people and other patients whilst providing a safe and therapeutic environment [10].

Aggressive and angry behavior may escalate in a predictable and orderly manner, thus providing opportunities for the healthcare professional to risk assess and intervene in the short-term though a process of de-escalation, thus avoiding confrontation. However aggression may also occur in a violent and abrupt pattern requiring emergency management and a coordinated staff response. The use of risk assessment methods helps determine the best intervention in order to reduce the potential for violent behavior. Also, the effectiveness of risk assessment lies in its advantage of reducing many un-necessary coercive measures like: seclusion, chemical and mechanical restraint [11].

In mental health services what is commonplace across international frontiers is that to prevent aggressive patients from harming themselves, other patients or staff, coercive measures and foremost, violence management strategies are required. By definition, coercion is in contravention to autonomy and informed choice by forcing a patient to undertake a course of action over which they have little or no control [12]. There is no agreement, recommendations or direction from the EU on which measures of coercion should be practiced across EU member states, and there is no overall one best practice approach. Violence management and coercive measures are very contentious, in particular given the variance in the management of violence across individual countries of Europe. For example; Steinert et al. [13] reported on opposing trends in the use of coercive interventions during mental health treatments in Germany and the Netherlands. Interestingly, in both countries, as in other EU states, ethical questions around areas of mental health have been a constant source of ongoing public debate. In a large study conducted of 77,681 nurses from ten European countries, the results identified an unacceptable level of violence from patients/relatives and considerable variance between countries with the highest rates reported for France, United Kingdom and Germany and the lowest rates for Norway and the Netherlands [14].

The European Working Conditions Survey, (EWCS) [15] included 44,000 workers from 34 countries. The survey enquired about verbal abuse; threats and humiliating behavior; physical violence; unwanted sexual attention; bullying and harassment; and sexual harassment encountered at work. Consistent with previous EWCS reports, the health sector, was one of the occupational groups at highest risk with rates of 16% for threats of violence and 15% for actual violence during the previous year, three times the respective 6 and 5% EU average.

In mental health services there are many methods used to manage violence and there is a broad literature on the use of coercive methods by mental health professionals. The predominant containment methods used in the management of violence and aggression include; physical restraint, seclusion rooms and pharmacological methods. Other action based activities focused on violence management include, risk assessment, observation, including staff vigilance and the use of CCTV, locked wards, increased staff patient ratios, de-escalation techniques and behavioral contracts. Mental Health Act legislation and, in particular, court mandated psychiatric treatment orders that require patients to accept psychiatric treatment with restrictive accompanying interventions will serve to reduce an individual’s movement and liberty. Court mandated patient admissions are becoming increasingly common and such patients are required to accept the mental health regimen, including restrictive movement and coercion. Mental health professionals are challenged on how to practice and research psychiatry within the current social and legal frameworks [12]. The attitudes of mental health workers towards their patients are important and listening to, and respecting the patient’s view may help to minimise any experience of coercion, even if the outcome is compulsory treatment.

There is very little evidence supporting the use of coercion in mental health and more worrying is the lack of evidence supporting one method of containment over another. There are some studies recommending that a systematic risk assessment/management approach could contribute to a reduction in the use of coercion [16, 17].

Muralidharan & Fenton [18] undertook a Cochrane review which compared the effects of various strategies used to contain acutely disturbed people during periods of psychiatric crisis. The authors reported that current non-pharmacological approaches to containment of disturbed or violent behavior are not supported by evidence from controlled studies. It was suggested that it is difficult to justify current methods of violence management, as practice is based on evidence that is not derived from well designed, conducted and reported randomized studies. In a systematic review, [19] no randomized controlled trials on coercion were identified, and based on case reports, case series cohorts it was concluded that patients believed that coercion was dehumanizing. It was recommended that health professionals should routinely consider that all patients have the potential to experience an intervention as coercive.

## Methods

The study emanated from an expert group, the European Violence in Psychiatry Research Group (EViPRG). (www.eviprg.eu). Members of this group embrace a mental health research agenda and there are members from 27 countries including non EU countries Australia, India and Canada. From the European EVIPRG members, the lead author of this paper (SC), invited at least one member from each country to act as the national project coordinator for their country. Agreements were obtained from 17 countries to participate in the study including: Ireland, England, Wales, Sweden, Netherlands, Germany, Norway, Switzerland, Denmark, Belgium, Turkey, Portugal, Romania, Iceland, Serbia, Bulgaria and Greece.

This study aimed to incorporate a European and multidisciplinary response in the determination of violence management practices and related research and education priorities across the 17 European countries. Given the international spread of respondents an eDelphi approach was selected for the study design and data collection. The Delphi technique seeks to gain consensus on the opinions of ‘experts’ through a series of structured questionnaires, [20] and experts communicate their opinions anonymously [21]. There are no universally agreed criteria for the selection of experts, and no guidance exists on the minimum or maximum number of experts on a panel; rather it appears to be related to common sense and practical logistics [22]. Experts in the clinical field may include clinicians, researchers and patients/lay people who have expertise by virtue of having experienced the impact of a condition or intervention [23]. Alternatively, it is suggested, [24] that rather than the term ‘expert’ one could use ‘knowledgeable participants’. In this study we defined ‘expert’ as those people working in mental health services interphasing with psychiatric patients/clients of the mental health services.

A key issue in using the Delphi technique is what percentage of agreement a researcher would accept as synonymous with consensus. However, the literature fails to provide clear guidelines on what level of agreement to accept [22]. Through a process of group discussion, consideration of the objectives of the study and a review of previous Delphi studies, a level of consensus at 70% was agreed for the study. Ethical approval was granted for the study by the Research Ethics Committee of the Royal College of Surgeons in Ireland (RCSI).

The eDelphi approach had been used previously, by the lead author, (SC), in an international priorities study [25]. In this study of violence management, a two-round eDelphi technique was used and data were collected using a commercially available online survey tool (http://www.surveymonkey.com). The tool was hosted off-site from the server of the host academic institution, with the questionnaire design and analysis of the full data set being available to one named administrator (EC).

A survey instrument was developed, agreed and validated through members of EVIPRG over two international meetings of the group. Members of EVIPRG include leaders and researchers in violence management, and at a convened meeting, members through the facilitation of the lead author of this paper (SC), agreed the content and structure and layout of the survey instrument. The National project coordinators translated the instrument into their native language, and when required with the assistance of fellow country members. As the instrument did not include any narrative or qualitative content and minimal wordage, back-translation was not conducted. Overall, there was a total of 14 languages.

The sampling approach to data collection was convenience with respondents self-selecting. Standard letters of introduction, study information and the URL link, in the native language of each country were then sent by the national coordinator to a cross-section of mental health professional and service organizations in the country. Each organization was requested to forward the email URL survey link to its individual members. Consent was obtained through the process of each individual on receipt of the email, could decide if they wished to participate by clicking on the URL link to the study provided in the email, therefore self-selecting themselves for the study. A limit to the number of participants was not set. This link included instructions on how to complete the study with a further link to the research ethics approval documents. Once the URL link was activated, the participant was directed to the study that had three screens (pages). The instrument included 12 questions arranged as: demographics, training, common interventions, education and research priorities, challenges, and self selection for round 2.

To encourage the identification of a wide array of views, the first round of the eDelphi study was qualitative in orientation, thus generating a large number of widely divergent statements [18]. Consistent with this principle, in round one, respondents were requested to list the greatest challenges for mental health professionals in managing violence and the priorities for education and the priorities for research in mental health. Reminders were sent after 30 and 60 day periods.

## Results

The results of round 1, when returned by the respondents to the administrator, (EC), were collated, and all email addresses and unique identifiers removed. The various lists of statements from participants, together with comments were then distributed to national coordinators for translation to English and cross-checking. When translated to English, the documents were returned to researchers, (SC, EC). Thematic content analysis of the data was commenced by two members of the research team (SC, JM) working independently, and jointly, using an established format [20]. In ensuring reliability, one of the national coordinators (AB), undertook a separate analysis of the Swedish data, which was the largest data set. This analysis subsequently was found to be consistent with the analysis undertaken by the other two members of the team. Data were analyzed by grouping similar items together. Where several items were identified to relate to the same issue, the researchers grouped them together to provide one universal description. The results of round one, including descriptors and groupings, were verified at a meeting of the EViPRG including the national project coordinators, so as to ensure agreement on analysis and representation of data.

The results included a total of 2809 respondents from 17 countries and 999 respondents who self-selected for round 2 eDelphi. The highest number of respondents were from Sweden 25.6% (*n* = 718) and the Netherlands 15.5% (*n* = 434), (Table 1). The professional role of respondents included a majority nurses, 57.3% (*n* = 1598), with Psychiatrists 10.6% (*n* = 295), (Table 2). The majority of respondents worked in acute psychiatry, 54% (*n* = 1511); outpatient departments, 10.5% (*n* = 295); and Forensic, 9.3% (*n* = 262). Other work areas of respondents include Rehabilitation, Primary Care and Emergency.

**Table 1:** Respondents location (*n* = 2809)

Sweden	718	25.6%
Netherlands	434	15.5%
Germany	319	11.4%
Norway	314	11.2%
Switzerland	227	8.1%
Denmark	136	4.7%
Ireland	128	4.5%
England	127	4.5%
Belgium	81	2.9%
Turkey	77	2.7%
Portugal	73	2.6%
Wales	45	1.6%
Romania	37	1.3%
Iceland	36	1.3%
Serbia	27	1.0%
Bulgaria	19	0.7%
Greece	11	0.4%

**Table 2:** Professional role of respondents (*n* = 2739)

Nurse	1598	57.1%
Psychiatrist	295	10.5%
Psychologist	195	7.0%
Administration	110	3.9%
Social Worker	109	3.9%
Occupational Therapist	62	2.2%
Counsellor	44	1.5%
Acute psychiatry in a psychiatric hospital	39	1.4%
Academic	24	0.9%
Physiotherapist	23	0.8%
Other therapists: (Psychotherapist, Cognitive Behavioural Therapist, and other country specific therapists)	290	10.8%

The distribution of years of experience for the overall population identified an experienced workforce with 42.4% of respondents qualified more than 16 years, and 21.5% qualified more than 25 years, (Table 3). However, across individual countries there was a contrasting pattern of results, with evidence of an ageing workforce in some countries. Results for the category more than 25 years qualified showed Ireland (34.4%); Sweden (33.6%); Germany (31%) and Bulgaria (31.6%). In other countries a much younger psychiatric workforce was profiled. Results in the category 0-5 years qualified showed the Netherlands (42.2%); Belgium (35.8%); Turkey (35.1%) and Norway (34.1%).

**Table 3:** Number of years qualified (*n* = 2809)

*0–5 years*	743	26.5%
*6–10 years*	463	16.5%
*11–15 years*	412	14.7%
*16–20 years*	291	10.4%
*21–25 years*	295	10.5%
*25 + years*	605	21.5%

It is of concern to note that, overall, 19.5% (*n* = 435) of respondents reported that they had not received training in the management of violence and aggression. Patterns of training varied across EU countries, and based on the number of responses to the question, the countries with the highest non training rates included; Romania; 88.5% (*n* = 23); Turkey 83% (*n* = 20); Portugal 44% (*n* = 25); Norway 32% (*n* = 75).

Participants were asked when they last received education and training in the management of violence and aggression, the majority reported that they had received training within the previous year, however, only 30% (*n* = 521) indicated that they had received training in the last year, with 9% (*n* = 157) reporting that it was longer than 5 years since training was received, (Table 4). In some countries it was reported that training had not been received for more than 5 years including: Bulgaria 85.7% (*n* = 6); Iceland 25% (*n* = 3); Norway 22.8% (*n* = 34).

**Table 4:** When respondents last received training (*n* = 1734)

*In the last 6 months*	666	38.4%
*In the last year*	521	30.0%
*In the last 5 years*	390	22.5%
*Longer than 5 years*	157	9.1%

Results showed, as might be expected, that the three most commonly used interventions in the management of violent patients were physical restraint 17% *n* = 943; seclusion 15% (*n* = 817); administering medications 14% (*n* = 795). Talk therapy 11% (*n* = 609), and de-escalation 7% (*n* = 393) were the next most commonly used interventions, (Table 5).

**Table 5:** Most commonly used interventions in the management of violence

Physical Restraint	943	17%
Seclusion	817	15%
Administering medications	795	14%
Talk therapy	609	11%
De-escalation	393	7%
Ensuring a safe environment	239	4%
Directing patients and care planning	227	4%
Observing behavior	191	3%
Risk Assessment	185	3%
Communications	182	3%
Diversional therapy	176	3%
Police Interventions	96	2%
Making agreement on patient violence	91	2%
Team work	86	2%
Other: Activate alarms; Discharge patients; ECT; More than 2 staff interventions, Security staff	390	7%
Non Category/unclear	176	3%

The three greatest challenges for mental health professionals identified by respondents included staff management and team work; competence and safety. Management support was rated as the 5^th^ greatest challenge, (Table 6).

**Table 6:** The greatest challenges for mental health professionals in managing violence

Staff Management staffing and Team work	400	14%
Competence	301	10%
Safety Protecting Staff & patients	266	9%
Education & training	199	7%
Management Support	185	6%
Risk assessment	170	6%
Coping, Fear & Abuse	161	6%
Preventing Violence & Violence Culture	137	5%
Clear Policy; Procedure & Protocol	131	5%
Physical Environmental/beds/facilities	107	4%
Reducing coercion	79	3%
De-escalation	78	3%
Ethical/Legal/Judiciary/Police Role	85	3%
Debriefing	68	2%
Alcohol and Drugs	60	2%
Other: Providing care; Funding and resources; Stigma; Seriously ill patients; Dementia.	170	6%
Non category/unclear	298	10%

Overall the study produced a total of 37,670 free text comments from 2494 respondents, (Table 7). Following translation and thematic content analysis of the data free text, comments were organized into a series of broad categories in preparation for round two. The second round of eDelphi was more specific, with the survey instrument seeking quantification of earlier findings, through ranking techniques to identify convergence in opinion [21]. During round 1, the levels of agreement with the questions on priority statements for education and research were summarized using mean, median and measures of dispersion, prior to round 2 circulation.

**Table 7:** Free text comments 37,670 provided by 2494 respondents

Country	5 common interventions	Challenges for professionals	Priorities for research	Priorities for education	Total comments
Sweden	1740	852	583	614	3789
Netherlands	1415	770	693	553	3431
Germany	932	522	375	421	2250
Norway	762	389	260	275	1686
Switzerland	50	458	315	336	1859
Denmark	545	280	190	175	1190
Ireland	437	331	258	220	1246
England	326	199	151	124	800
Belgium	248	141	119	105	613
Turkey	204	56	50	46	356
Portugal	209	109	82	77	477
Wales	136	99	53	50	338
Romania	82	53	41	42	218
Iceland	57	42	35	29	163
Serbia	69	47	41	29	186
Bulgaria	35	29	20	24	108
Greece	45	30	28	22	125
Total	7992	4407	3294	3142	18,835

Following round 1 analysis, the round 2 circulation list which included 40 education priorities and 40 research priorities, was sent via email back to respondents who consented for round 2 (*n* = 999). In an effort to gain consensus, individuals were invited to rank each statement individually on a seven-point Likert scale. Seven represented ‘top priority’, while 1 represented ‘not a priority’. Reminders were sent after 30 and 50 day periods to all participants.

The results for round 2 consensus on top priorities for education and top priorities for research in violence management are outlined in Tables 8 and 9. Similar items are consistently identified; most notably the top priorities included: preventing violence; the influence of environment and staff on levels of violence; best practice in managing violence; risk assessment and the aetiology and triggers for violence and aggression.

**Table 8:** Top priority areas for education in violence management (*n* = 999)

Best practice in managing violence & aggression	220	11%
Preventing violence	218	11%
Aetiology, triggers for violence & aggression	182	9%
Environment & staff influence on violence & aggression	150	8%
Risk Assessment	117	6%
Strategic Management of violence	99	6%
staff in-service training and effectiveness	92	5%
staff attitudes to violence and coping skills	92	5%
De-escalation and Breakaway	84	4%
Incidence/prevalence/statistics	72	4%
Staffing and skill mix	63	3%
Pharmacotherapy	61	3%
Service user experience/involvement	56	3%
Substance abuse and violence	65	3%
Effects of violence on staff/clients	44	2%
Debriefing and aftermath of violence	50	2%
Staff-Patient relationships	42	2%
Diagnosis and violence	33	2%
Other: Communication; teamwork; legislation and policy; economics costs; ethics and rights; ethnicity; elderly and children aggression, safety and security, mechanical restraint, managing violence in community care	150	8%
Non category/unclear	64	3%
Total	1954	100%

**Table 9:** Top priority areas for research in violence management

Influence of environment and staff attitudes on levels of violent behavior	270	11%
Preventing violence	269	11%
Best practice in the management of violence and aggression	233	10%
Risk Assessment	181	7%
Understanding aetiology and triggers for violence & aggression	174	7%
Causes/diagnosis/definitional issues	150	6%
Legislation strategic and policy in violence management	143	6%
De-escalation and Breakaway	114	5%
Effectiveness of staff training	91	4%
Service user experience and involvement	83	3%
Coping and aftermath of violence	82	3%
Use of pharmacotherapy	78	3%
Incident reporting/prevalence/statistics	75	3%
Staffing and skill mix	71	3%
Substance abuse and violence	65	3%
Effects of violence on staff and clients	54	2%
Debriefing	45	2%
Staff-Patient relationships	43	2%
Communication	36	1%
Collaboration and Teamwork	35	1%
Comparing violence management methods	26	1%
Other: Violence in community care; economic costs; ethnicity; forensic violence; child aggression; influence of media; ethics and rights.	98	4%
Total	2416	100%

## Discussion

The central hypothesis of this study, is that within the European region there is no clear direction or uniformity on best practice in the management of violence across mental health services. The enlargement in the number of countries in the European Union in recent years has further exacerbated the lack of commonality in areas of service development, standards, education and the role of the mental health professionals. Freedom of movement, conflicts and complex emergencies have created a large number of refugees and asylum seekers across Europe. Such migrants include vulnerable people who are at an increased risk of psychosocial trauma following acts of ethnic cleansing, murder, sexual violence, torture and mutilation. Europe at the moment is struggling to find ways forward in the ongoing humanitarian catastrophe of refugees and asylum seekers and as part of any response to the crises European mental health services must be prepared to meet needs, in terms of violence prevention and management. It may be suggested that refugees and asylum seekers are an evolving mental health grouping requiring dedicated resources for services and specialist staff training and researching.

From an EU perspective, the validity of the findings of this large scale study is supported by the spread of countries, from across the EU, and the opinions of respondents from all the major professions involved in the delivery of mental health services. A major benefit of this study is the richness of the results which provides us with consensus on the significant issues that are confronting mental health professionals in the EU, at present, and into the future.

Whereas the majority of respondents were from Western Europe, the inclusion of respondents from Turkey, Bulgaria and Serbia is a rather unique, but important addition. As the majority of mental health professionals in Europe are nurses, it was an expected finding that nurses would form the largest group of respondents. Generally, other professionals as respondents are proportionate to numbers employed in a typical mental health service, (Table 2). There has been discussion about the ageing work force in Europe [26] and this study has identified a pattern of an ageing workforce in mental health services. The ageing mental health staff profile should be of concern, as this study identified that 21.5% of respondents were qualified more than 25 years. The pattern of older age staff profile is particularly evident in Ireland, Sweden and Germany. The creation of a European Mental Forum to review skill requirements with manpower planning is a realistic objective.

There is a vast mental health literature, mostly non-empirical, on assault and violent behavior and the importance of education and training in violence management. However, the impact of such literature is dubious, given the sustained increase in assaultive behavior and violence and a major failing appears to be the translation of training to the practice setting. Many authors have commented on the absence of an empirical evidence base on which to base either the training or practice of violence prevention, [27] In this study the lack of an EU directive on training in violence management and aggression is reflected in the unacceptably high proportion of respondents who had never received education and training and those who had not received it in the previous 5 or more years.

Definitions of the term coercion apply to a wide range of interventions and involve the use of authority to override the choices of an individual [28]. The range of interventions can vary from a highly restrictive use of force to more delicate interpersonal skills [29]. Historically, coercion has been a cornerstone of violence management across a Europe dominated by large mental institutions with a custodial care model. The more traditional methods of violence management used over generations are dominant in the results of this European study, with physical restraint, seclusion and medication as the main reported interventions. Talk therapy was identified as an intervention, and was manifest as verbal de-escalation, and as a primary preventive intervention when the patient is not upset and as a post incident follow up talk.

There is a strong tradition of institutional care in Europe and primarily, because of this, it may be argued that mental health professionals are trapped within an institutional model, which adversely affects the type and level of service that they can provide. In the early 1970s, governments across Europe became increasingly concerned about the large mental institutions and the custodial nature of mental health services. A Mental Health Declaration for Europe and a Mental Health Action Plan were endorsed by the Ministers of Health of the 52 member states in the European region of the WHO in 2005. The Declaration favoured community-based mental health care settings. However such a utopian aspiration is hampered by the lack of a single and unifying ideological approach to mental health services in Europe, and a prevailing dominant country national policy approach, which arguably contributes to ideological eclecticism across Europe [2].

A greater understanding of the mental health challenges common to all countries must serve to drive developmental initiatives to determine the nature and scope of professional practice in Europe. In this study, it is interesting that management issues rather than clinical issues are dominant in the results of the greatest challenges, with staff management and team work identified as the major challenge. This finding in consideration with other top challenges identified such as management support; clear policy, procedure and protocol, raises concerns about leadership and management and may suggest the need for change in how mental health services are managed and governed, and for employers/service providers to be more strategic in planning service developments.

A key factor in planning any service is to determine the education and research priorities of the professionals who are accountable for providing the services. The respondents from across all professional disciplines expressed a strong theme for education in violence prevention. Top priorities for education and research include violence prevention, risk assessment, and the aetiology and triggers of violence and aggression. The agreement on priorities among all mental health professionals supports the case for multidisciplinary training in violence prevention and management. The influence of the environment of care, staffing levels and best practice in managing violence are priorities. There is sufficient evidence available to suggest that the culture of the clinical environment of care can influence the development of violent incidents. Since 2000 and most recently, [30] the Royal College of Psychiatrists UK, in recognizing that the clinical environment may be a factor that influences the development of violent incidents, developed guidelines on appropriate general layout and structure of the clinical environment. The intention of the guidelines is to provide direction to mental health professionals on a number of elements in the clinical environment that serve to influence and trigger the development of violent incidents. The extent to which any initiatives have been implemented and evaluated is unclear.

There are some limitations to the study and these in the main relate to translation. When translating and adapting instruments across languages, there is no guarantee that the different language versions are equivalent. Back-translation was not conducted, however in this study, reliability is introduced by the fact that all translators were bilingual and that the content to be translated were lists of words, rather than long narrative passages. Whereas the use of online methods of enquiry are economic and efficient, the lack of the traditional relationship between the researchers and the respondent may impact on ability to encourage a response.

## Conclusion

The increased concern among employees, employers and professional organizations over the escalation in violence and aggression has created an urgent requirement for proactive national and international strategies in safety and violence prevention. Despite improved initiatives in areas such as risk assessment, and staff training there remains a paucity of readily available data regarding the safety of mental health services.

Despite the growth in legislation and in the number of Health & Safety Authorities in various EU member states, in many European countries there is an alarming lack of clarity on matters of procedure and policy pertaining to ward safety and security in psychiatric hospitals. Across the psychiatric services there is a bewildering array of practices ranging from an open door policy to locked wards and doors and confiscation of patients clothing and personal property, some of which borders on the infringement of human rights, liberty and the rights and choices of patients [10].

Psychiatric services across Europe appear to be fragmented with no identified ideological position and there are few links, with little sharing, comparisons or collaborative research. In Europe, language differences are a reality and may have contributed to insular thinking, however, it must not be seen as a barrier to sharing best practice. There is a need to grow a formal communication mental health communication network in Europe.

Historical accounts of mental health services, document a prominent evolving role for staff in violence management in the traditional psychiatric hospital setting. However there is now a greater emphasis on the use of risk assessment methods, and this helps to determine the best intervention in order to reduce the potential for violent behavior, and reduce unnecessary coercive measures. However, European standards for violence management in mental health can only be achieved through political initiatives, promulgated at parliamentary level and engaging a stakeholder model. Central to discussions are the regulatory, professional, patient-representative and legal obligations of health authorities and employers in ensuring best practice in violence management for the benefit of patients and staff.

Arising from this study a number of actions are recommended:

- The publication of position papers on violence management from the EU parliament to advice governments, employers and appropriate professional bodies in each EU member state;
- Establishment of a European forum which includes stakeholders to develop, agree and publish best practice guidelines on violence management;
- Establishment of EU directives on risk assessment and staff training in the management of workplace violence;
- The availability of EU funding for research on best practice in violence management.

## Abbreviations

EU: European union
EViPRG: European violence in psychiatry research group
EWCS: European working conditions survey
